# p150 ADAR1 isoform involved in maintenance of HeLa cell proliferation

**DOI:** 10.1186/1471-2407-6-282

**Published:** 2006-12-08

**Authors:** Haifang Wang, Zheng Hou, Yumei Wu, Xue Ma, Xiaoxing Luo

**Affiliations:** 1 Department of Pharmacology, School of Pharmacy, the Fourth Military Medical University, Xi' an 710032, P. R. China; 2 Current affiliation: Faculty of Life sciences, Northwestern Polytechnical University, Xi’an 710072, P. R. China

## Abstract

**Background:**

RNA-specific adenosine deaminase ADAR1 is ubiquitously expressed in a variety of mammalian cells and tissues. Although its physiological importance in non-nervous tissues has been confirmed by analysis of null mutation phenotypes, few endogenous editing substrates have been identified in numerous peripheral tissues and biological function of ADAR1 has not been fully understood.

**Methods:**

A conditional site-specific, ribozyme-based gene knock-down strategy was utilized to study the function of full-length isoform of ADAR1 (p150 protein) in HeLa cell. Double-stable HeLa cell lines were developed by transfecting HeLa Tet-On cells with a pTRE-derived plasmid that can express a hammerhead ribozyme against mRNA of p150 ADAR1 isoform under induction condition. Semi-quantitative RT-PCR and Western blotting were performed to measure the expression of p150 in selected cell clones. Cell proliferation was evaluated by means of MTT assay and growth curve analysis. Cellular morphological changes were observed under light microscope. Flow Cytometry was used for cell cycle analysis. Growth rate of cell transplants in BALB/c nude mice was also investigated.

**Results:**

Both HeLa cell proliferation in vitro and the growth rate of transplanted HeLa cell-derived tumors in nude mice in vivo were significantly inhibited due to reduced expression of ADAR1 p150. Additionally, cell cycle analysis showed that cell progression from G1 phase to S phase was retarded in the ADAR1 p150 suppressed cells.

**Conclusion:**

Our results suggest that normal expression and functioning of p150 ADAR1 is essential for the maintenance of proper cell growth. The mechanisms underlying ADAR1's action might include both editing of currently unknown double-stranded RNAs and interacting with other cellular dsRNA-related processes.

## Background

Adenosine (A) to inosine (I) conversion catalyzed by adenosine deaminases acting on RNA (ADAR) is an evolutionally conserved process, occurring in organisms from unicellular protozoa to humans [1,2]. Through site-specific modification of pre-mRNAs, this process provides an important post-transcriptional mechanism for expanding the functional diversities of RNA and protein products, such as those that encode the glutamate receptors and serotonin receptors [3,4]. In contrast, nonspecific editing is more frequent and has been considered mainly implicated in host defense mechanisms [5].

Due to the use of different promoters, human ADAR1 has two major variants: The full-length form p150 is expressed from an interferon (IFN) -inducible promoter and predominantly localizes to cytoplasm. Use of a constitutive promoter, in contrast, produces an N-terminally truncated, predominantly if not exclusively nuclear p110 protein that is not induced by IFN [6,7]. Owing to its special cytoplasmic location and elevated deaminating activity during infection, IFN-inducible p150 is believed to play a role in antiviral defense against viruses that replicate in the cytoplasm [8,9].

The physiological importance of ADAR1 has been confirmed by phenotypic analysis of null mutants in previous studies. Inactivation of ADAR1 gene has been reported to cause lethality in ADAR1^+/- ^chimeric mice and ADAR1^-/- ^embryos, along with severe impairment of liver structure and reduced hepatocyte cell density [10-12]. The remarkable observation that among many ADAR1 deleted embryonic tissues that underwent apoptosis, the highest level of apoptosis was within the liver and regions exhibiting excessive apoptosis in ADAR1^-/- ^embryos corresponded to tissues expressing the highest levels of ADAR1 in wild-type embryos indicated an essential role of ADAR1 in the normal development of non nervous tissues [10]. Moreover, compared with wild-type MEF (mouse embryonic fibroblasts) cells, ADAR1^-/- ^MEF cells were prone to apoptosis when subjected to serum deprivation, and expression of ADAR1 p150 increased significantly in wild-type MEF cells during serum deprivation [12], perhaps indicating a special requirement of ADAR1 p150 for cell survival.

Recently, Samuel and his colleagues reported that small amount of ADAR1 p150 could be detected in almost all tissues of healthy adult mice [13]. Expression of p150 was found to be least abundant in brain tissue of normal mice, and this seems somewhat correlated with the previous observation that no abnormality was detected in ADAR1-deleted embryonic brain [10-12]. They also found that p150 is most abundant in liver of mice infected with Salmonella and where it is also the major form of ADAR1. Taken together, these data suggest that endogenous ADAR1 p150 protein may play a critical role in the normal development and maintenance of bodily tissues, especially for liver. However, despite the ubiquitous expression of ADAR1 and the correlated large amount of I-containing mRNA (I-mRNA) throughout the body [1,14,15], as of yet, only a few genes with editing sites in coding regions have been identified as endogenous substrates of ADAR1 [3,4,16].

The expression and regulation of ADAR1 p150 in human tissues has not been fully characterized thus far. In HeLa cells, the simultaneous presence of two forms of ADAR1 has been confirmed by both Western-blotting and immunofluorescence analysis [17,18]. In the present study, by using a method based on hammerhead ribozyme technique, we specifically knocked down the expression of mRNA for p150 ADAR1 isoform in HeLa cells and first revealed that endogenous p150 is necessary for the proper control of cell proliferation.

## Methods

### Design and construction of recombinant vector

Based on the previously reported method [19], a hammerhead ribozyme was designed to target the human ADAR1 mRNA (Genbank ACCESSION NM_001111.3 GI: 7669471) at position 206, which is included in coding sequence of p150 ADAR1 protein but absent in that of p110 protein. Consequently, cellular phenotypes associated with induced-expression of this specific ribozyme would be absolutely caused by deficiency of p150 ADAR1 isoform. The designed ribozyme oligonucleotide 5'- TGAGGGAA CTGATGAGTCCGTGAGGACGAA ACCCCTGC 3' (The underlined are nucleotides complementary to the substrate) and the corresponding reverse complementary strand were synthesized and annealed after heating to 100°C for 2 min followed by a slow cooling to room temperature. For inducible ribozyme expression in mammalian cells, the double-stranded DNA fragments were subcloned into the Tet-On pTRE plasmid (Clontech, Palo Alto, CA, USA) to form a vector designated as pTRE-ADAR1-Rz, which would release free ADAR1-Rz transcripts in the presence of doxycycline (Dox). The integrity of the constructs was confirmed by enzymatic digestion and DNA sequencing.

### Cell cultures and stable transfection

HeLa Tet-On cells (Clontech, Palo Alto, CA, USA) were cultured in DMEM medium (Gibco, Gaithersburg, MD, USA) supplemented with 10% fetal bovine serum, 100 IU/mL penicillin and 100 μg/mL streptomycin. Cells grown in 10 cm dishes were co-transfected with pTRE-ADAR1-Rz and pTK-Hyg plasmids, or pTRE and pTK-Hyg plasmids, using Lipofectamine 2000 (Gibco, USA) transfection method. Transfected cells were screened for hygromycin (Hyg) resistance. The selected clones were designated as HeLa-Rz cells (carrying both pTRE-ADAR1-Rz and pTK-Hyg) or HeLa-p cells (carrying both pTRE and pTK-Hyg). Positive clones were further analyzed for the expression of p150 ADAR1 isoform in the absence/presence of Dox at a final concentration of 2 μg/ml by using RT-PCR and Western blotting methods, respectively.

### RNA isolation and RT-PCR

Total RNA was extracted using the TriZol reagent (Invitrogen, Carlsbad, CA, USA) and reverse transcribed using random hexamer primers and reverse transcriptase (Superscript II, Invitrogen) at 43°C for 1 h. The cDNAs prepared with RNAs obtained from different cell clones were PCR-amplified using *Taq *DNA polymerase (Invitrogen) and the following ADAR1 specific primer pairs: 5'- AGAAGGGCAAGCTACAGA -3' (nucleotides 742–759) and 5'- AATTCAGGGCAGAGGAG -3' (nucleotides 1136–1152) that flank exons 1–2 and capable of detecting transcripts of ADAR1 p150 (Genbank ACCESSION NM_001111.3 GI: 70166851) only. β-actin was also PCR-amplified and used as an internal control. The PCR step was performed for 23–32 cycles of denaturizing at 94°C for 60 s, annealing at 55°C for 1 min, primer extension at 72°C for 1 min, and a final extension at 72°C for 10 min. PCR products were analyzed by gel (1% agarose) electrophoresis analysis.

### Western blotting

Total protein extracts of cultured cells obtained by using Trizol reagent was prepared in SDS sample buffer and used for Western-blotting analysis [17]. Samples containing 25 μg of total protein were fractionated by SDS-PAGE on a 10% acrylamide gel and transferred onto a nitrocellulose membrane. ADAR1 immunoreactivity was detected using rabbit antiserum specific for the human ADAR1 protein (provided by Dr. Michael F. Jantsch of Department of Chromosome Biology at University of Vienna) as primary antibodies. Detection of antigen-antibody complex formation was with HRP (horseradish peroxidase) – conjugated goat anti-rabbit secondary antibody using an ECL detection kit (Amersham Biosciences, Piscataway, NJ).

### Viability assessment

Cell viability was assessed using 3-(4, 5-dimethylthiazol-2-yl)-2, 5-diphenyl tetrazolium bromide (MTT) dye conversion. Following Dox (2 μg/ml) exposure, MTT was added to the wells to a final concentration of 1 mg/mL and incubated at 37°C for 2 h. The reaction products were solubolized overnight using 50 % dimethylformamide/20 % sodium dodecyl sulfate, and 96-well plates were read on a plate reader (Molecular Probes, Sunnyvale, CA, USA) at 590 nm. Viability was determined by comparison to untreated cells. Cells were counted daily with a Coulter Counter (Beckman Coulter, Fullerton, CA, USA). Mean cell count data were then used to construct a growth curve and the population doubling time was calculated as reported previously [20]. The assessments were conducted in triplicate.

### Flow cytometry analysis

1 × 10^6 ^cells were harvested and resuspended in 100 μl of the kit reaction buffer containing 5 μg/mL of propidium iodide. After mixing, cells were incubated for 15 min in the dark. Cell cycle analysis was performed on a Model Coolper XL cytofluorimeter and analyzed by a Multicycle Software. Experiments were run in triplicate.

### Growth of cell transplants

BALB/c nu/nu nude mice aged 6–8 wks were obtained from the Shanghai Institute of Materia Medica at the Chinese Academy of Sciences in China. The experimental protocol was approved by the Experimental Animal Center of Chinese Academy of Sciences (Identification No. Scfk11-6A-0006). HeLa-Rz cells were grown in monolayer culture, harvested, washed twice, resuspended in Hank's Balanced Salt Solution and implanted into the dorsal subcutaneous tissue of mice by injection at 0.2 mL (1 × 10^7 ^tumor cells for each animal). Animals were then randomly divided into two groups: the ADAR1 knockdown group (given drinking water containing 2.5 mg/mL of Dox) and the control group. The tumor size was measured twice a week with calipers and tumor volume was estimated by the formula: (length × width^2^)/2 [21].

### Statistical analysis

Data are expressed as mean ± standard deviation (SD). Statistical comparisons were made using unpaired Student's t-tests. A difference was considered significant at a value of *P *< 0.05.

## Results

**Figure 1:**
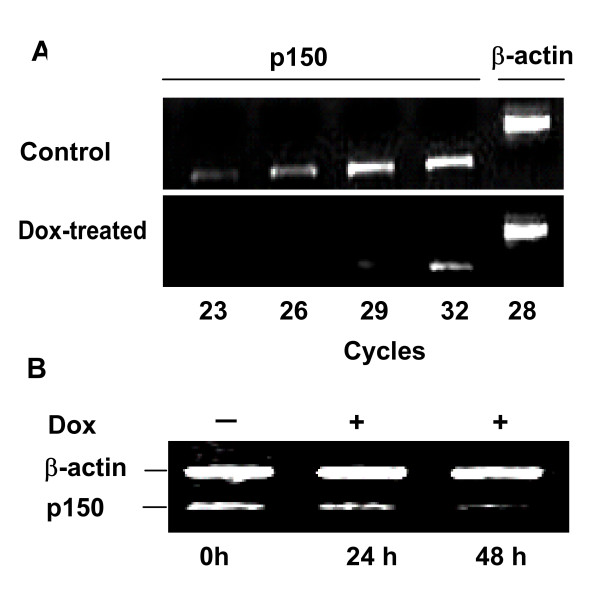
**A. Effect of Dox induction on expression of mRNA for p150 isoform of ADAR1 in HeLa-Rz cells. B. Time-dependent knockdown of ADAR1 p150 gene in HeLa-Rz cells by Dox induction**. RNA was isolated from HeLa-Rz cells treated with 2 μg/mL of Dox or left untreated and then was analyzed by RT-PCR. PCRs were carried out using primers that flank exons 1–2 of human ADAR1 mRNA (representing p150 isoform) or β-actin plus and minus primers, respectively. PCR products were analyzed by gel electrophoresis after the indicated number of amplification cycles.

**Figure 2:**
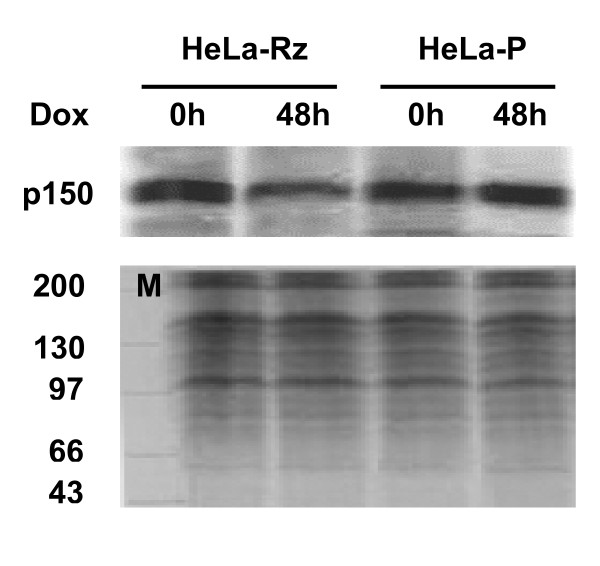
**Effect of Dox induction on expression of p150 ADAR1 isoform in HeLa cells**. Western blotting analysis of ADAR1 protein expression was performed on HeLa-Rz cells and HeLa-P cells treated with 2 μg/mL of Dox or left untreated respectively using methods described in the related text.

HeLa Tet-On cells transfected with the constructed pTRE-ADAR1-RZ or the control carrier were named as HeLa-Rz and HeLa-P cell clones, respectively. A total of 24 HeLa-Rz cell clones were collected and measured for Dox-induced alteration in expression of mRNA of p150 ADAR1. The relative abundance of ADAR1 p150 was similar for all cell clones in the absence of Dox (data not shown). Addition of 2 μg/ml of Dox significantly reduced the level of mRNA of p150 ADAR1 isoform in a few HeLa-Rz cell clones, and the inhibitory effect was in a time-dependent manner (Fig. 1). The result of Western blotting analysis was shown in Fig. 2: p150 isoform of ADAR1 was detected in both HeLa-Rz cells and HeLa-P cells by the antibody we used. After Dox treatment, the amount of p150 protein was decreased significantly in HeLa-Rz cells, in contrast, expression of p150 in HeLa-p cells was nearly not affected.

**Figure 3:**
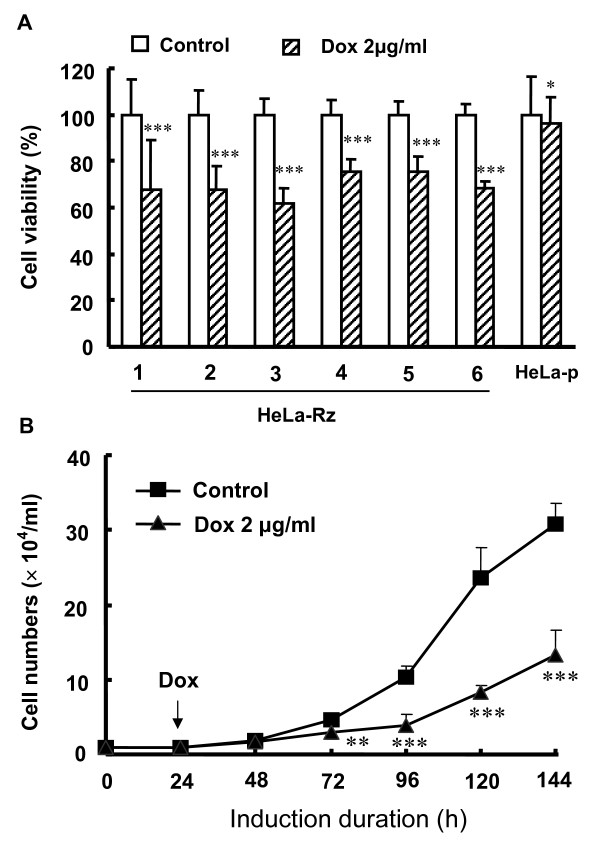
**A. Effect of p150 isoform of ADAR1 knockdown on cell viability**. Cells were cultured in the absence/presence of Dox for 72 h. 1~6: Different HeLa-Rz cell lines. HeLa-p cell line was used as a control (n = 10). * *P *> 0.05, ** *P *< 0.05, ***P < 0.01 *vs *control. **B. Effect of Dox on growth curve of HeLa-Rz cells. **Cells were grown in media containing 2 μg/mL of Dox and the media was changed every two days. Data are expressed as mean ± SD (n = 6), *P > 0.05, ** *P *< 0.05, ***P < 0.01 *vs *control.

To reveal the potential correlations between level of p150 ADAR1 isoform and cellular biological properties, we examined cell viability of different HeLa-Rz cell clones in response to Dox treatment. The results showed that, in addition to the decrease of p150 expression, relative cell viability of HeLa-Rz clones was also reduced (with an inhibitory rate in the range of 25% to 38.0%) by three days of Dox induction, whereas the viability of HeLa-p cells was not affected by Dox treatment (Fig. 3A). A Dox-induced downward shift in the cellular growth curve was observed in a HeLa-Rz cell clone (shown in Fig. 3B). The calculated population doubling time (Gt) (21.52 ± 1.20 h) of HeLa-Rz cells was prolonged (32.43 ± 3.40 h, P < 0.01) significantly by Dox treatment. However we did not observed obvious morphological changes under the light microscope.

**Figure 4:**
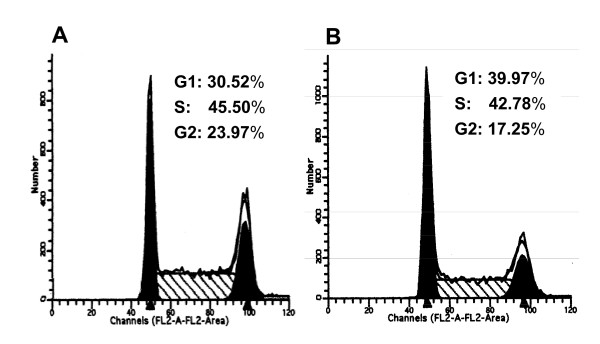
**Cell cycle analysis of HeLa-Rz cells**. Propidium iodide staining was performed and samples were analyzed by flow cytometry. **A**. Cell cycle of untreated cells. **B**. Cell cycle is altered after 48 h of Dox (2 μg/mL) treatment.

HeLa-Rz cells were grown in medium containing 2 μg/mL Dox for 48 h, causing the expression level of ADAR1 mRNA to decrease. Flow cytometry analysis detected a constant increase in G_1 _DNA content (from 38.87 ± 0.11 % to 46.53 ± 0.10 %, p < 0.05) in comparison with the control group. The percentage of cell DNA in replication phase S slightly decreased, but statically the change was not significant (39.94 ± 0.08 % *vs *37.49 ± 0.07 %, p > 0.05). These data suggest that Dox treatment, acting through knockdown of the p150 ADAR1 mRNA, could lead to an arrest of cells in G_1 _phase, a delayed transition into S phase, and eventually restrained proliferation of cells (Fig 4). Under our experimental condition, Dox did not induce apoptosis.

**Figure 5:**
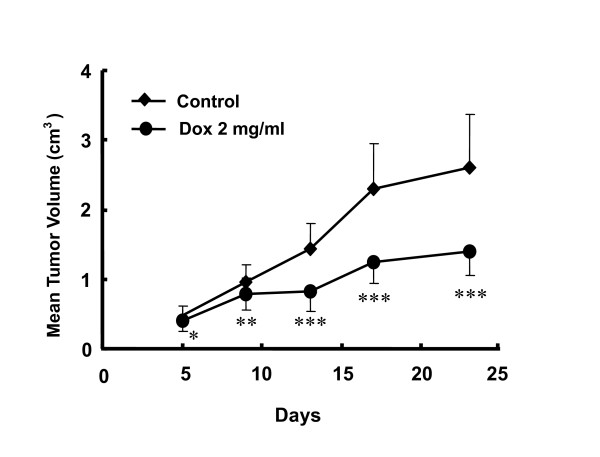
**Growth rate of HeLa-Rz cell transplants**. Nude mice received subcutaneous injection of 1 × 10^7 ^HeLa-Rz cells and randomly grouped into the control or Dox treatment groups (2.5 mg/mL Dox in drinking water throughout the entire experimental period) (n = 8 for each group). * *P *> 0.05, ** *P *< 0.05, ***P < 0.01 *vs *control.

A single HeLa-Rz cell clone was picked out based on its high sensitivity to Dox induction and the cells were cultured. Nude mice were given subcutaneous injections of cultured HeLa-Rz cells and the growth rate of cell transplants was monitored. At 5 days post inoculation, all animals developed a palpable tumor at the injection site, indicating the cell tumorigenecity was not altered. However the rate of tumor growth in the Dox-treated group was significantly slower compared with the control group (Fig. 5). This difference became detectable (P < 0.05) by day 11. By day 23 the mean tumor size in Dox-treated mice was 1.38 ± 0.34 cm^3^, whereas in the control group it had reached 2.60 ± 0.69 cm^3^, which corresponding to an about 60 % inhibitory rate of tumor growth. The health of the mice was not affected as evaluated by the lack of body weight loss. Taken together, these data indicate that decrease of p150 ADAR1 isoform significantly inhibited growth of transplanted HeLa-Rz cells, but does not affect tumor induction in nude mice.

## Discussion

The sequences of ADAR1 from human, rat, and mouse are highly conserved, all having a unique N-terminus (containing two tandemly arranged Z-DNA-binding domains), three double-stranded RNA-binding domains (dsRBDs) and a conserved deaminase domain at the C-terminus [6]. Its distinct structural architecture, tightly regulated expression and intracellular distribution set ADAR1 apart from other known ADAR gene family members, ADAR2 and ADAR3 [22-24]. Furthermore, the discrepancy between its widespread expression within the body and the few known substrates indicates that ***in vivo ***function of ADAR1 may not be restricted to nuclear pre-mRNA editing [13,15,16].

Due to its interferon-inducible expression and its predominantly cytoplasmic localization, p150 isoform of ADAR1 has been considered involved in host defense mechanisms against viruses that replicate in the cytoplasm [8,9]. More recently, the observation that serum deprivation significantly increased cytoplasmic level of p150 expression in MEF cells suggests that p150 might also play a role critical for the promotion of cell survival [11].

In the present study, by using RT-PCR and Western blotting methods, we demonstrated that endogenous p150 isoform (full-length isoform of human ADAR1) of ADAR1 is expressed abundantly in HeLa cells. In order to study its biological function, we specifically down regulated the level of mRNA for p150 protein by using a hammerhead ribozyme-based technique. Our results revealed that a several fold knockdown of ADAR1 p150 inhibited cell proliferation (by a maximum inhibitory rate of 38%) significantly. In addition, cell cycle progression of p150 suppressed cells was characterized by an excessive retention of cells in G_1 _phase and delayed transition into S phase. However, we did not find any obvious cellular morphological changes under the light microscope. In nude mice, the growth rate of transplanted HeLa cells was also significantly reduced by down-regulation of ADAR1 p150 expression.

The molecular mechanisms underlying the action of p150 ADAR1 protein to maintain cellular proliferation is still under investigation. In vitro, the enzyme can act on almost any double stranded substrate of sufficient length, showing little site selectivity [25]. Secondly, because any RNA that is at least partially double-stranded represents a potential substrate for A-to-I editing, ADAR1 may interfere with other cellular processes that rely on dsRNA molecules, such as RNAi [26] and NF90 proteins (including NF110, NF90 and NF45) [23]. Finally, supported by the observation that both expression and activity of ADAR1 p150 are elevated in lymphocytes in response to inflammation [27], it appears that cytoplasmic editing activity of ADAR1 p150 might also be involved in the regulation of gene expression under special pathological conditions.

Based on our study, endogenous expression of ADAR1 p150 protein is necessary for the proper control of cell growth. p150 protein is also capable of responding to "unfavorable stimulations" and thus involved in host defense mechanisms through its inducible expression, cytoplasmic accumulation and shuttling activity between the nuclear and cytoplasmic compartments [6,9,17,22]. Recent bioinformatics analysis has revealed that A-to-I editing modification occurs in at least 30 different organs with different frequencies and most editing events are found within Alu elements embedded in the non-coding regions of human transcriptome, which is suggested to be engaged in the modulation of tumor cell growth and differentiation [28-32]. Further investigation is required to address the questions whether and which form of ADAR1 is associated with tumor development.

## Conclusion

Our results suggest that normal expression and functioning of p150 ADAR1 isoform is essential for the maintenance of proper cell growth. The mechanisms underlying the action of ADAR1 p150 might include both cytoplasmic editing of currently unknown dsRNAs and interacting with other cellular dsRNA-related processes.

## Competing interests

The author(s) declare that they have no competing interests.

## Authors' contributions

HW participated in the design of the study, performed plasmid construction, cell culture, cell transfection and RT-PCR analysis, and drafted the manuscript, ZH performed Western blotting analysis, YW and XM participated in the animal experiment and performed statistical analysis. XL conceived of the study, participated in its design and coordination and revised the article critically for important intellectual content. All authors read and approved the final manuscript.

## Pre-publication history

The pre-publication history for this paper can be accessed here:
